# Uterine Dysfunction in Diabetic Mice: The Role of Hydrogen Sulfide

**DOI:** 10.3390/antiox9100917

**Published:** 2020-09-26

**Authors:** Emma Mitidieri, Domenico Vanacore, Carlotta Turnaturi, Raffaella Sorrentino, Roberta d’Emmanuele di Villa Bianca

**Affiliations:** 1 Department of Pharmacy, School of Medicine, University of Naples, Federico II, Via D. Montesano, 49, 80131 Naples, Italy; emma.mitidieri@unina.it (E.M.); domenico.vanacore@unina.it (D.V.); carlotta.turnaturi@unina.it (C.T.); demmanue@unina.it (R.d.d.V.B.); 2 Department of Molecular Medicine and Medical Biotechnologies, School of Medicine, University of Naples, Federico II, Via Pansini, 5, 80131 Naples, Italy; 3 Interdepartmental Centre for Sexual Medicine, University of Naples, Federico II, Via Pansini 5, 80131 Naples, Italy

**Keywords:** contraction, diabetes, hydrogen sulfide, 3-mercaptopyruvate-sulfurtransferase, non-obese diabetic mice, spontaneous motility, uterus

## Abstract

It is well-known that the physiological uterine peristalsis, related to several phases of reproductive functions, plays a pivotal role in fertility and female reproductive health. Here, we have addressed the role of hydrogen sulfide (H_2_S) signaling in changes of uterine contractions driven by diabetes in non-obese diabetic (NOD) mice, a murine model of type-1 diabetes mellitus. The isolated uterus of NOD mice showed a significant reduction in spontaneous motility coupled to a generalized hypo-contractility to uterotonic agents. The levels of cyclic nucleotides, cAMP and cGMP, notoriously involved in the regulation of uterus homeostasis, were significantly elevated in NOD mouse uteri. This increase was well-correlated with the higher levels of H_2_S, a non-specific endogenous inhibitor of phosphodiesterases. The exposure of isolated uterus to L-cysteine (L-Cys), but not to sodium hydrogen sulfide, the exogenous source of H_2_S, showed a weak tocolytic effect in the uterus of NOD mice. Western blot analysis revealed a reorganization of the enzymatic expression with an upregulation of 3-mercaptopyruvate-sulfurtransferase (3-MST) coupled to a reduction in both cystathionine-β-synthase (CBS) and cystathionine-γ-lyase (CSE) expression. In conclusion, the increased levels of cyclic nucleotides dysregulate the uterus peristalsis and contractility in diabetic mice through an increase in basal H_2_S synthesis suggesting a role of 3-MST.

## 1. Introduction

Diabetes mellitus (DM), either type-1 diabetes mellitus (T1DM) or type-2 diabetes mellitus (T2DM), is a lifelong condition that affects millions of individuals worldwide [1,2], and it represents a strong risk factor for the development of atherosclerotic coronary and peripheral arterial disease [3,4]. It has been reported that changes in the balance of hydrogen sulfide (H_2_S) play an important role in the pathogenesis of β-cell dysfunction that occurs in response to T1DM and T2DM [5,6]. H_2_S is synthesized by mammalian tissues, and it serves various important regulatory functions [7,8,9]. It is endogenously produced in mammalian cells from the amino acid L-cysteine (L-Cys) through the activation of two pyridoxal-5-phosphate-dependent enzymes, i.e., cystathionine-β-synthase (CBS) and cystathionine-γ-lyase (CSE) or 3-mercaptopyruvate-sulfurtransferase (3-MST). Both CSE and CBS are expressed within the pancreas, and H_2_S is involved in glucose homeostasis [5]. Indeed, H_2_S production and signaling are altered during both T1DM and T2DM [6,10,11,12,13]. In vivo and in vitro studies suggest that an excess of H_2_S in pancreatic islets could be involved in both T1DM and T2DM [14]. In vivo, in streptozotocin-induced diabetes, a T2DM model, the levels of CBS and CSE increase in both the pancreas and liver as well as in islet β cells of diabetic animals [15,16]. In addition, both CBS and CSE are expressed in insulin-secreting pancreatic β cells and the H_2_S produced inhibits insulin secretion by activating ATP-sensitive potassium channels [14]. In this scenario, whether this increase in islet of H_2_S biosynthesis represents a protective mechanism or it is involved in the pathophysiology of the disease is not clearly defined. On the other hand, in diabetic patients, the plasmatic levels of H_2_S are significantly lower as compared to the normal subjects [17].

Despite the fact that numerous studies have described the role of H_2_S in complications associated to diabetes, little is reported on the contribute of H_2_S in genitourinary tract disorders and particularly in uterine homeostasis in non-pregnant condition in diabetes.

With the onset of T1DM menstrual cycle disturbance, subfertility, earlier menopause, as well as pregnancy complications have been described [18,19,20,21]. T1DM is thought to disrupt the physiological morphology of the myometrium and then the normal uterus functionality [22,23,24]. It is well known that the physiological uterine peristalsis, related to several phases of reproductive functions, plays a pivotal role in fertility and female reproductive health [25,26,27]. Indeed, the correct alternation between contractions and periods of quiescence helps ova and sperm transport directs the embryo to its implantation site and contributes to the expulsion of menstrual debris [27,28]. Poorer contractility, in terms of amplitude and duration, has been observed in uteri from diabetic patients compared with normoglycemic subjects. This reduction in force has been associated with a reduction in muscle content, smooth muscle cell myofibrils, and calcium channel receptors and signaling in myometrial tissues [22,29]. Other reports show that diabetes affects not only the morphology of the myometrium but also its normal function such as the contractile response of the myometrium to oxytocin stimulation [30].

H_2_S modulates uterus contraction. Its tocolytic effect is mainly driven by the CSE-derived H_2_S as demonstrated by experiments performed on CSE^-/-^ mice [31]. Non-obese diabetic (NOD) mice are a consolidated murine model of TDM1 that is characterized by i) an impairment of endogenous H_2_S biosynthesis at vascular level ii) a reduction in H_2_S plasmatic levels that paralleled to the severity of disease [12] and iii) a uterine weight loss involving both the endometrium and the myometrium [32]. Alterations in H_2_S signal could be causative for the onset of uterus disorders such as oligomenorrhea, premature menopause, polycystic ovarian syndrome that can interfere with the female reproductive health occurring in diabetic patients. Therefore, in order to better clarify the role played by H_2_S pathway in changes driven by diabetes, we investigated the possible mechanism responsible for the alteration of uterine contractions in NOD mice aiming to evaluate the involvement of H_2_S signaling.

## 2. Materials and Methods

### 2.1. Animals

**Figure 1:**
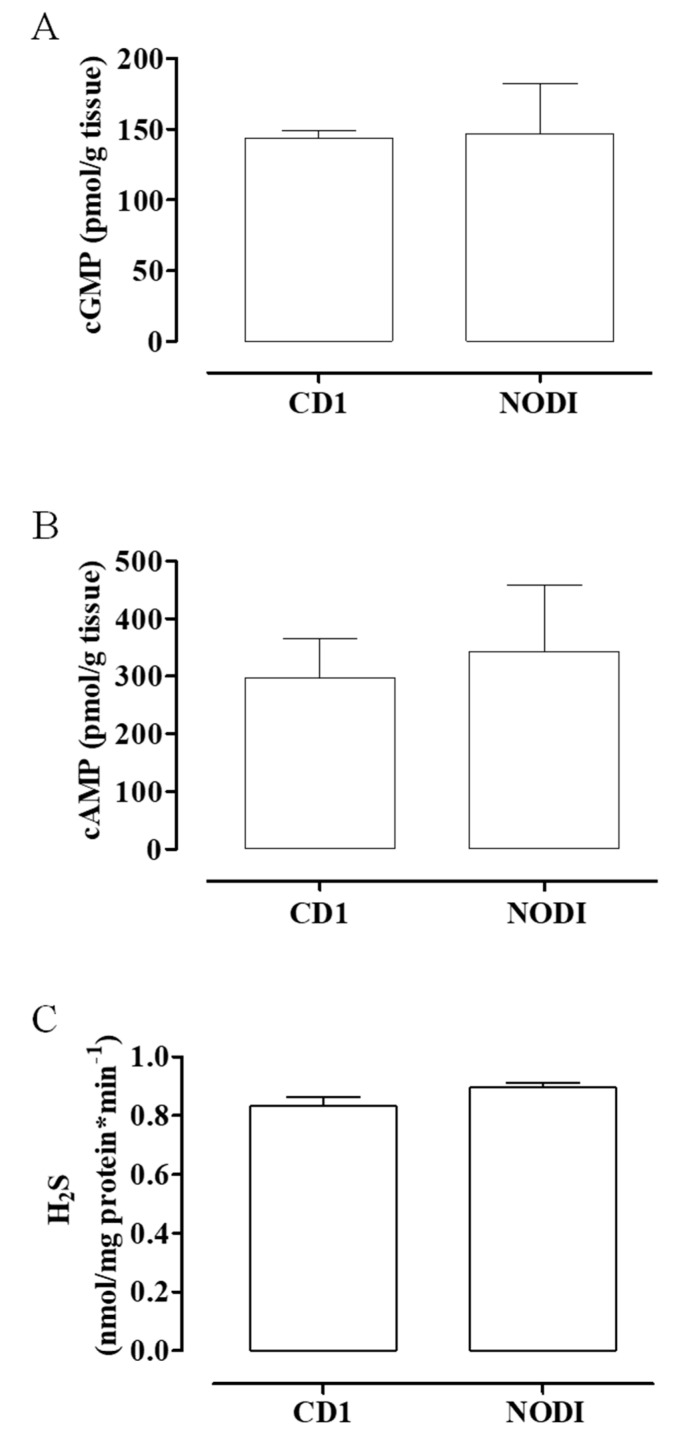
cGMP, cAMP, and H_2_S levels in CD1 and NODI mouse uteri. The basal levels of cGMP (**A**), cAMP (**B**), and H_2_S (**C**) were not significantly different between CD1 (control mice (CTR)) and NODI mice. NODI mice have normal glucose levels.

NOD mice represent a strain with an elevated susceptibility in developing T1DM [33]. These mice show changes with the evolution of pathology; in particular, there is an early phase characterized by localization of inflammatory cells, such as T cells and activated macrophages, around the pancreatic islet, inducing peri-insulitis (4–10 weeks of age); consequently, these cells infiltrate islets and initiate progressive destruction of pancreatic β cells, resulting in a drastic reduction in insulin plasma levels (12–30 weeks of age). The progression of diabetic pathology and its clinical outcomes in these animals are similar to those in humans and associated with vascular disorders. NOD mice are classified as NODI in which diabetic state is not yet present, NODII has glycosuria and elevated glycaemia, and NODIII displays a severe pathology with even higher levels of glycosuria and glycemia [34]. In a preliminary set of experiments, CD1 control mice showed a similar profile with NODI mice (Figure 1A–C); for this reason and in line with the literature [34,35], this work was carried out on NODIII mice and age-matched CD1 control mice (CTR) (Charles River, Italy).

Diabetes was assessed through the measurement of glycemia (monitored weekly) by applying a drop of blood to a chemically treated, disposable ‘test-strip’, which is then inserted into an electronic blood glucose meter. When glycemia values were higher than 500 mg/dL, mice were euthanized and named NODIII. Virgin female mice were kept at temperatures of 23 ± 2 °C, humidity range 40–70% and 12 h light/dark cycles. Food and water were provided ad libitum. All animal care and experimental procedures in this study were performed according to the Declaration of Helsinki (European Union guidelines on the use of animals in scientific experiments) and the ARRIVE guidelines, and the study was authorized by national and local animal care office (Italian Health Minister and Centro Servizi Veterinari, Università degli Studi di Napoli Federico II-CD9CF-N.NCQ).

### 2.2. Organ Bath Studies

Mice were euthanized during the estrus period and uteri, rapidly dissected, and cleaned of fat and connective tissue, and were placed in a dish containing Krebs’ solution [115.3 mM NaCl; 4.9 mM KCl; 1.46 mM CaCl2; 1.2 mM MgSO4; 1.2 mM KH2PO4; 25 mM NaHCO3; 11.1 mM glucose (Carlo Erba, Milan, Italy)].

The uteri, harvested from both CTR and NODIII mice, were dissected and divided into two horns. Each horn was cross-cut into two strips and mounted in an isolated organ bath containing oxygenated (95% O2 and 5% CO2) Krebs’ solution at 37 °C, as previously described [31]. Tissues, connected to isometric transducers (FORT25, World Precision Instruments, 2Biological Instruments, Besozzo VA, Italy) associated to Power Lab 8/35 (2Biological Instruments, Besozzo VA, Italy), were stretched until a resting tension of 0.3 g. After 30 min of equilibration, when the homogeneous spontaneous contractility was observed, the response to acetylcholine (Ach, 10 µM, Sigma, Milan, Italy), oxytocin (Oxy, 0.005 U/mL, Sigma, Milan, Italy), or prostaglandin F2α (PGF2α, 0.1 µM, Sigma, Milan, Italy) was evaluated. In another setting of experiments, on stable spontaneous contractility, a concentration–response curve of L-Cys (100 nM–300 μM) or sodium hydrogen sulfide (NaHS, 100 nM–300 μM) was obtained. Data were calculated as frequency (peaks/min or % of spontaneous motility) or as force (g or dyne/mg tissue). Results were expressed as the mean ± SEM (*n* = 6 mice) and analyzed by using analysis of variance (ANOVA) followed by Bonferroni post hoc test or unpaired Student’s *t*-test as needed. *p* < 0.05 was considered significant.

### 2.3. H_2_S Determination

H_2_S production was measured in NODIII and CTR mice in samples of horn uterus homogenates, as previously described [36]. The samples were lysed in a modified potassium phosphate buffer (100 mM, pH 7.4, sodium orthovanadate 10 mM, and proteases inhibitors). Protein concentration was determined by using the Bradford assay (Bio-Rad Laboratories). Homogenates were added to a reaction mixture containing pyridoxal-5′-phosphate (2 mM), L-Cys (10 mM) or vehicle. H_2_S production was measured in presence of vehicle corresponds to the basal values and took into account the contribution of all three enzymes; the addition of L-Cys to the homogenates caused an increase in H_2_S production derived from CBS and CSE activity. In another setting of experiments, the inhibitors of H_2_S biosynthesis, DL-propargylgicine (PAG, 10 mM, CSE inhibitor), aminoxiacetic acid (AOAA, 1 mM, CBS inhibitor), or a combination of both were added 5 min before addition of L-Cys in uterus homogenates. The reaction was performed in sealed Eppendorf tubes and initiated by transferring tubes from ice to a water bath at 37 °C for 40 min. Next, the trichloroacetic acid solution (TCA, 10% wt/vol) was added to each sample followed by zinc acetate (1% wt/vol). Subsequently, N,N-dimethyl-p-phenylendiamine sulfate (DPD; 20 mM) in HCl (7.2 M) and FeCl3 (30 mM) in HCl (1.2 M) were added, and optical absorbance of the solutions was measured after 20 min at a wavelength of 668 nm. All samples were assayed in duplicate, and H_2_S concentration was calculated against a standard curve of NaHS (3–250 μM). Data were calculated as nmol/mg protein*min-1. Results were expressed as mean ± SEM (*n* = 6 mice, experiments performed in the presence of inhibitors) or mean ± SEM (*n* = 10 mice, experiments performed in the absence of inhibitors). Data were analyzed by one-way ANOVA followed by Bonferroni post-test. *p* < 0.05 was considered significant.

### 2.4. Western Blot Analysis

Western blot was performed on samples of horn uterus harvested from NODIII mice or CTR mice as previously described [37]. Samples were homogenized in modified RIPA buffer (50 mM Tris-HCl pH 8.0, 150 mM NaCl, 0.5% sodium deoxycholate, 0.1% sodium dodecyl sulfate, 1 mM EDTA, 1% Igepal) (Roche Applied Science, Italy) and protease inhibitor cocktail (Sigma-Aldrich, USA). Protein concentration was determined by Bradford assay using albumin (BSA) as standard (Sigma-Aldrich, USA). Denatured proteins (50 μg) were separated on 10% sodium dodecyl sulfate-polyacrylamide gels and transferred to a polyvinylidene fluoride membrane. The membranes, blocked in PBS containing 0.1% *v/v* Tween 20 and 5% non-fat dried milk for 1 h at room temperature, were incubated with mouse monoclonal antibody for CSE (1:1000; Abnova, Milan, Italy), rabbit polyclonal for CBS (1:1000; Santa Cruz Biotechnology, Inc.), or rabbit polyclonal for 3-MST (1:500, Novus Biologicas, Cambridge, UK) overnight at 4 °C. Membranes were washed in PBS containing 0.1% *v/v* Tween-20 and then with horseradish peroxidase-conjugated secondary antibody for 2 h at room temperature. Next, membranes were extensively washed and developed using Chemidoc (Biorad, Milan, Italy). The target protein band intensity was normalized over the intensity of the housekeeping protein β-actin (1:5000, Sigma-Aldrich, Milan, Italy). Data were calculated as OD*mm2 as optical density (OD)*mm2. Results were expressed as mean ± SEM (*n* = 8 mice) and analyzed by unpaired Student’s *t*-test. *p* < 0.05 was considered significant.

### 2.5. cGMP and cAMP Measurement

Samples of horn uterus harvested from both CTR and NODIII mice were dropped into 5–10 vol (mL buffer/g tissue) of TCA (5%) and homogenized by using a polytron-type homogenizer. Samples were centrifuged at 1500g for 10 min and cyclic guanosine monophosphate (cGMP) or cyclic adenosine monophosphate (cAMP) were measured in supernatants as described in the manufacture’s protocol of cGMP and cAMP EIA Kit (Cayman, Vinci Biochem, Vinci, Italy) [38,39]. All samples were assayed in duplicate, and cyclic nucleotide concentrations were calculated against a calibration curve of standard cGMP or cAMP. Data were calculated as pmol/g tissue. Results were expressed as mean ± SEM (*n* = 6 mice) and analyzed by unpaired Student’s *t*-test. *p* < 0.05 was considered significant.

## 3. Results

### 3.1. Diabetes Strongly Reduces Uterine Spontaneous Motility

**Figure 2:**
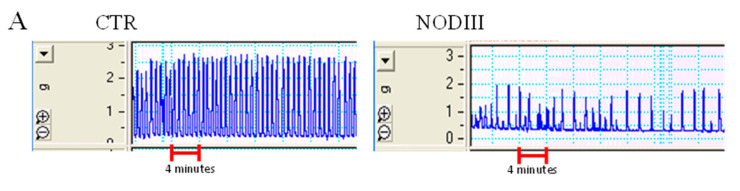

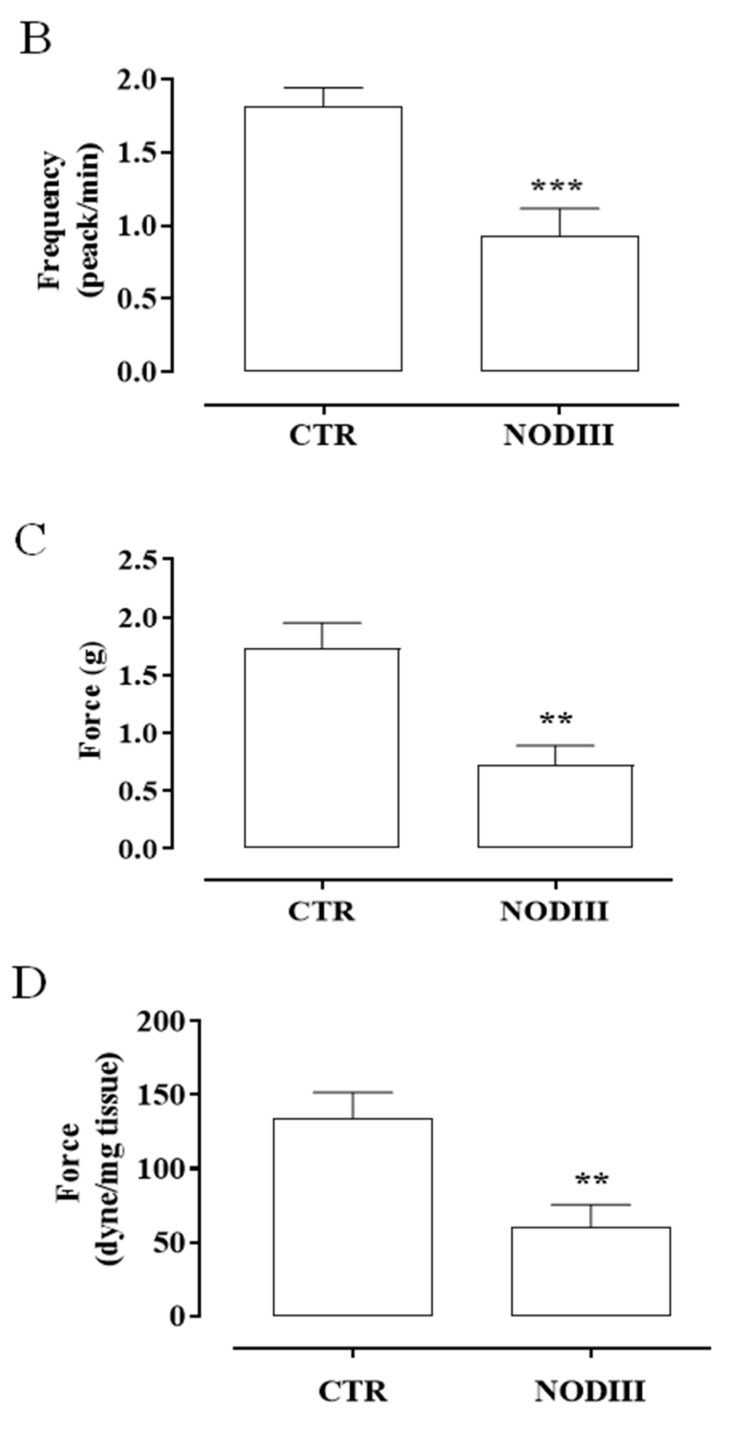
Spontaneous contractility in NODIII mice. A typical tracer of the basal contractility signal recorded by uteri harvested from CTR or NODIII mice (**A**). The basal response of uteri harvested from NODIII mice is significantly reduced compared to CTR mice. Data are reported as frequency, i.e., peak/min (**B**) (*** *p* < 0.001); contraction force, expressed in grams (panel **C**; ** *p* < 0.01); or as dyne/mg tissue (**D**) (** *p* < 0.01). Results are calculated as mean ± SEM (*n* = 12 mice) and analyzed by unpaired Student’s *t*-test.

As showed in the typical tracer in Figure 2A, uteri harvested from NODIII mice show a reduction in the frequency (peak/min) of spontaneous motility compared to CTR mice. The frequency of spontaneous motility is significantly reduced in NODIII mice compared to CTR mice (Figure 2B; *** *p* < 0.001).

### 3.2. Diabetes Strongly Reduces Uterine Spontaneous Contractility

In the same setting of experiments, the contraction force of spontaneous contraction has been evaluated. The force of contraction expressed as gram (g) is significantly reduced in the uterus of NODIII mice compared to CTR mice (Figure 2C, ** *p* < 0.01). This effect is also showed in the typical tracer (Figure 2A). In order to ascertain this finding, the force of contraction has been also evaluated taking into account the weight of uterus. The horn uterus weight is significantly (* *p* < 0.05) reduced in NODIII mice of about 30%, i.e., CTR 15.85 ± 2.5 (*n* = 12 mice) vs. NODIII 9 ± 0.4 mg (*n* = 12 mice). Because of the significant difference in uterus weight, the data on contractility have been expressed also as dyne/mg tissue. The contraction force is significantly lower in NODIII mice compared to CTR (Figure 2D; ** *p* < 0.01). 

### 3.3. Isolated Uterus of NODIII Mice Displays an Impaired Response to Different Contracting Agents

**Figure 3:**
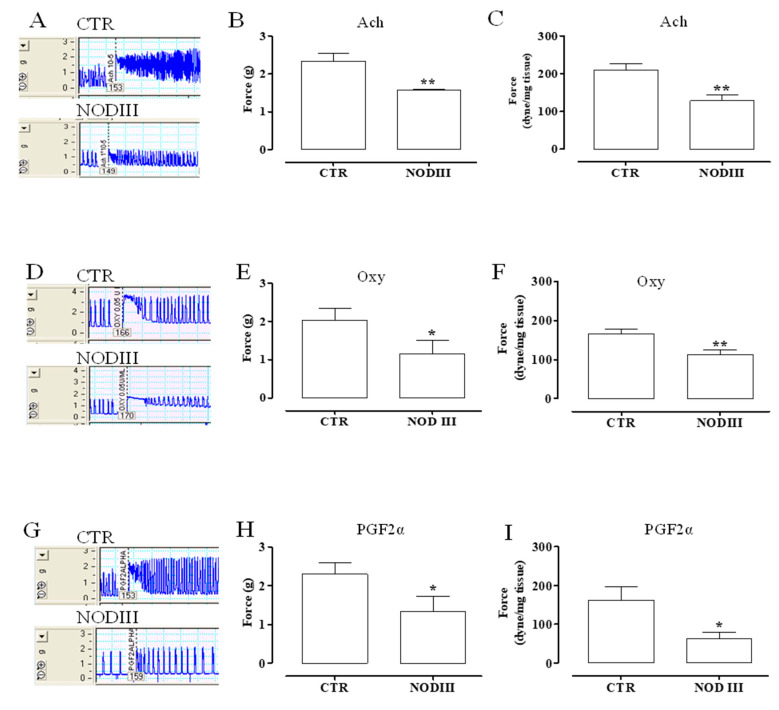
Uterine response to contracting agents in NODIII mice. (**A**) shows a typical tracer of Ach-induced contraction of uteri harvested from either CTR or NODIII mice. The contractile response to Ach (10 µM) is shown as gram (**B**) or dyne/mg tissue (**C**). It is significantly reduced in NODIII mice vs. CTR mice (** *p* < 0.01). (**D**) shows a typical tracer of Oxy-induced contraction of uteri harvested from CTR or NODIII mice. The contractile response to Oxy (0.005 U/mL) is reported as gram (**E**) or dyne/mg tissue (**F**), and it is significantly reduced in NODIII mice vs. CTR mice (* *p* < 0.05, ** *p* < 0.001, respectively). (**G**) shows a typical tracer of PGF2α-induced contraction of uteri harvested from CTR or NODIII mice. The contractile response to PGF2α (0.1 µM) is shown as gram (**H**) or dyne/mg tissue (**I**), and it is significantly reduced in NODIII mice vs. CTR mice (* *p* < 0.05). Results are calculated as mean ± SEM (*n* = 6 mice) and analyzed by unpaired Student’s *t*-test.

Typical tracers of the isolated uterus response to Ach, Oxy, or PGF2α are reported (Figure 3A,D,G). The contraction has been evaluated as force of contraction and expressed as g and dyne/mg tissue (Figure 3). The contractile response to Ach is significantly reduced in NODIII mice in terms of both g and dyne/mg tissue (Figure 3B,C, respectively; ** *p* < 0.01). Oxy-induced effect on uterus motility is also significantly reduced compared to CTR mice (Figure 3E,F; * *p* < 0.05; ** *p* < 0.01). Similarly, PGF2α contraction is significantly lower in NODIII mice compared to CTR mice (Figure 3H,I; * *p* < 0.05).

### 3.4. The Content of cAMP and cGMP Is Higher in Uteri Harvested from NODIII Compared to CTR Mice

**Figure 4:**
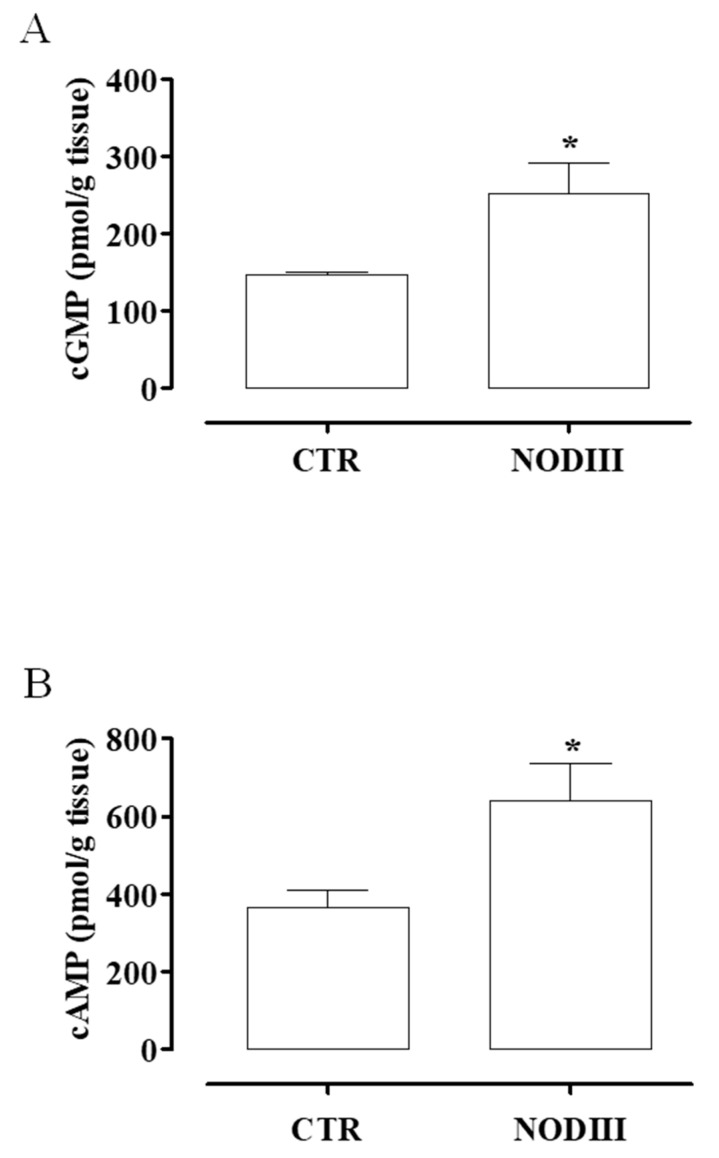
Changes in cAMP and cGMP content in uteri of CTR and NODIII mice. The uterus content of cGMP (**A**) or cAMP (**B**) is significantly increased in uteri harvested from NODIII mice vs. CTR mice (* *p* < 0.05). Results are expressed as pmol/g tissue and calculated as mean ± SEM (*n* = 6 mice). Results are analyzed by unpaired Student’s *t*-test.

To evaluate the possible mechanism involved in the dysmotility of the diabetic uterus, cyclic nucleotides have been evaluated. The content of cGMP (Figure 4A) and cAMP (Figure 4B) is significantly increased in the uterus of NODIII mice (* *p* < 0.05). The levels of both cyclic nucleotides are increased by 2 fold over the control values.

### 3.5. Diabetes Interferes with H_2_S Pathway

**Figure 5:**
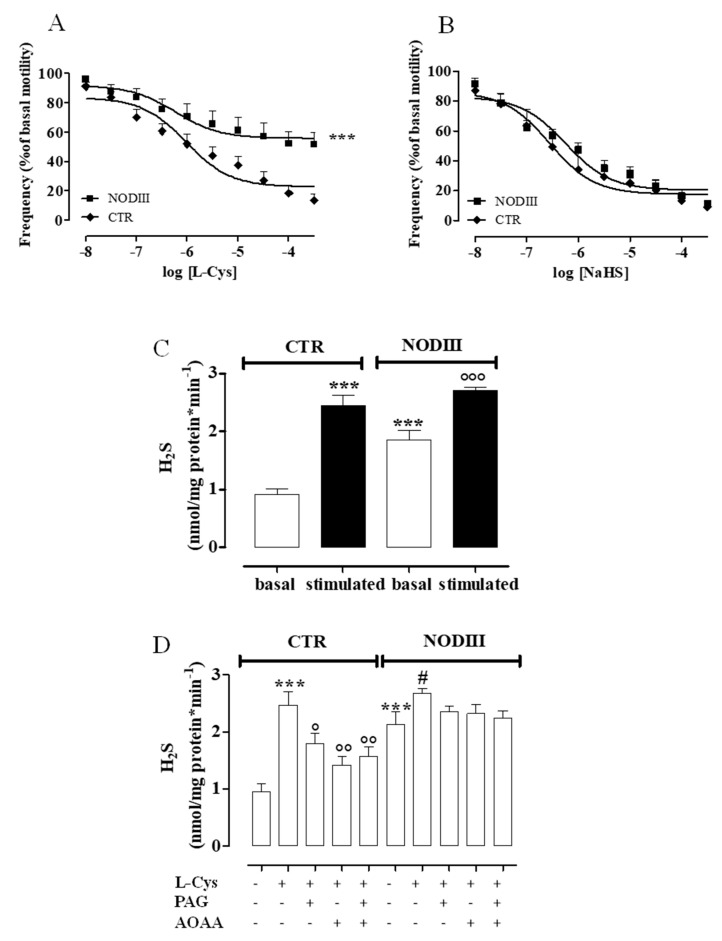
Changes in H_2_S pathway in uteri of CTR and NODIII mice. The tocolytic effect of L-Cys (10 nM–300 µM) is significantly reduced in NODIII mice vs. CTR mice (**A**) (*** *p* < 0.001). The tocolytic effect of NaHS (10 nM–300 µM) does not significantly differ between NODIII and CTR mice (**B**). Results are expressed as frequency (% of basal motility) and calculated as mean ± SEM (*n* = 6 mice) and analyzed by using analysis of variance (ANOVA) followed by Bonferroni post hoc test. The basal amount of H_2_S is significantly increased in NODIII mice vs. CTR mice (*** *p* < 0.001). H_2_S production induced by L-Cys is significantly increased in CTR mice (*** *p* < 0.001) or in NODIII mice (°°° *p* < 0.01) vs. the respective basal values (**C**). Results are expressed as nanomoles per milligram of protein per minute and calculated as mean ± SEM (*n* = 10 mice). Results are analyzed by one-way ANOVA followed by Bonferroni post-test. L-Cys significantly increases H_2_S production in both CTR and NODIII mice uterus (*** *p* < 0.001 and # *p* < 0.05, respectively). DL-propargylgicine (PAG) (10 mM, CSE inhibitor), aminoxiacetic acid (AOAA) (1 mM, CBS inhibitor), or their combination were added before L-Cys (10 mM) challenge in uterus homogenates. The incubation with inhibitors significantly reduced the increase in H_2_S production induced by L-Cys in CTR mice (° *p* < 0.05 and °° *p* < 0.01) but not in NODIII mice (**D**). Results are expressed as nanomoles per milligram of protein per minute and calculated as mean ± SEM (*n* = 6 mice). Results are analyzed by one-way ANOVA followed by Bonferroni post-test.

To assess the role of H_2_S, a concentration–response curve of L-Cys (10 nM–300 µM), the endogenous substrate for H_2_S production, has been performed on horn uteri harvested from both NODIII and CTR mice (Figure 5A). Interestingly, the tocolytic effect induced by L-Cys is significantly reduced in NODIII compared to CTR mice (Figure 5A; *** *p* < 0.001). NaHS (10 nM–300 µM) induces a tocolytic effect at the same extent in NODIII and CTR mice (Figure 5B). Unexpectedly, the basal amount of H_2_S in diabetic uteri is 2-fold and significantly higher when compared with CTR mice (Figure 5C; *** *p* < 0.001). The evaluation of enzymatic ability, i.e., CBS and CSE in the uterus has been tested by incubating the homogenized tissue samples with L-Cys. The amount of H_2_S produced is significantly higher as compared to the respective control (Figure 5C; ***, °°° *p* < 0.001). The inhibition of either CBS or CSE significantly reduces the H_2_S production induced by L-Cys in homogenized tissue obtained from CTR but not from NODIII mice (Figure 5D; ° *p* < 0.05; °° *p* < 0.01).

### 3.6. Diabetes Alters the Expression of CBS, CSE, and 3-MST

**Figure 6:**
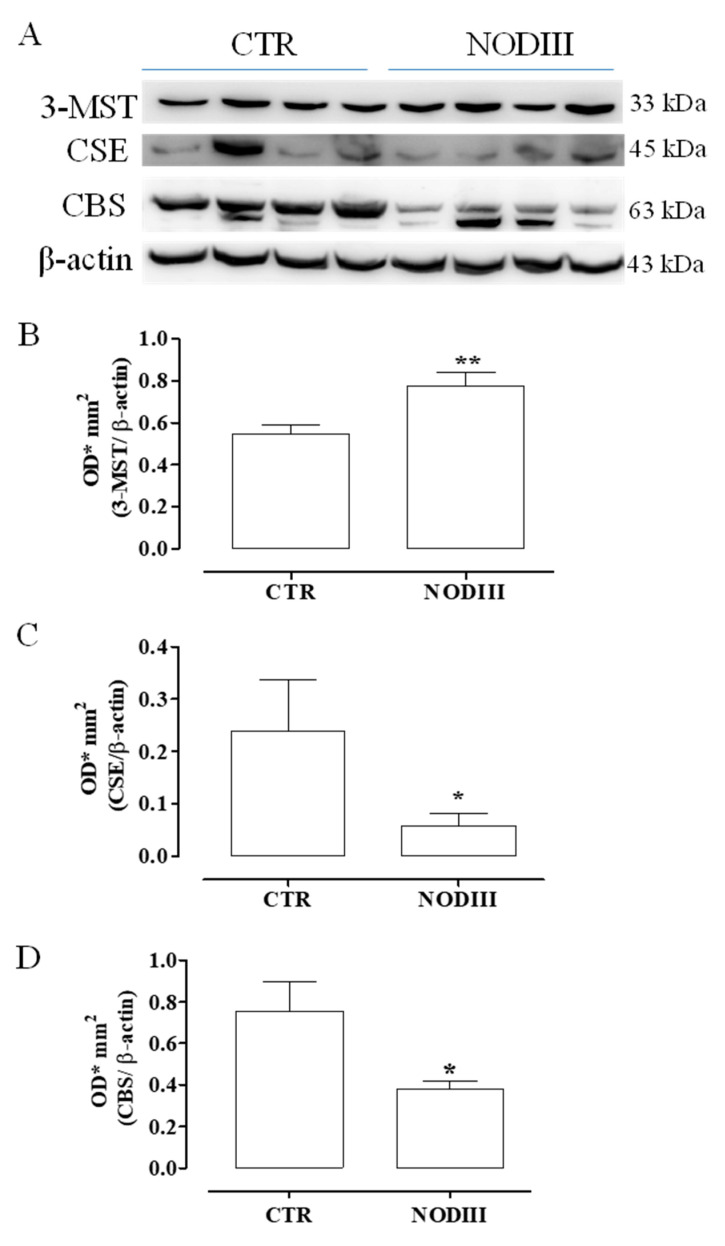
Expression of 3-MST, CSE, and CBS in uteri of CTR and NODIII mice. Representative western blot for 3-MST, CBS, and CSE (**A**). Expression of 3-MST is significantly higher in NODIII mice uteri vs. CTR mice (**B**) (** *p* < 0.01). CSE expression is significantly reduced in NODIII mice vs. CTR mice (**C**) (* *p* < 0.05). CBS expression is significantly reduced in NODIII mice vs. CTR mice (**D**) (* *p* < 0.05). Results are normalized against β-actin as housekeeping protein and calculated as mean ± SEM (*n* = 8 mice). Data were calculated as OD*mm2 as optical density (OD)*mm2. Results are analyzed by unpaired Student’s *t*-test.

Western blot analysis clearly shows that the expression of 3-MST is significantly higher in the uterus of in NODIII mice (Figure 6B; ** *p* < 0.01). Conversely, CBS and CSE expression are significantly reduced in NODIII mice (Figure 6C,D; * *p* < 0.05).

## 4. Discussion

Diabetes mellitus (DM), encompassing T1DM and T2DM, one of the most common noninfectious progressive and chronic diseases worldwide, is liable for deleterious effects in many organs and systems. The disease development leads to microvascular and macrovascular complications that alter organ perfusion with significant changes also in the reproductive system [2,40]. However, alteration of the uterus functionality in female diabetic patients is an under-investigated complication [41,42,43,44]. The intensification of insulin therapy and the improvement of metabolic control ameliorate menstrual and reproductive disorders, although they do persist [18,45]. Despite improvements in diabetes therapy patients still face abnormalities in their pubertal development, menstrual cycles, fertility, and age of menopause, with hyperandrogenism and oligomenorrhoea being the most prevalent problems in young adult DM patients. Alterations in the structure, biochemistry, and innervation of the uterus may lead to the dysregulation of the correct alternation of contractions and periods of quiescence [27]. The present study takes into account this lack of information about uterus functionality in diabetes in non-pregnant condition. Therefore, the research has been carried out by using NOD mice, a mouse model for long term T1DM, where female mice exhibit the pathophysiological features of human T1DM [23]. Indeed, considering the high levels of glycemia, hypoinsulinemia, duration of diabetes, and the alteration of myometrium morphology and function, NODIII mice reproduce the characteristics of long-term DM in women [22]. Poor myometrial contractility has been demonstrated in vitro in women with DM and gestational diabetes [29]. In line with these findings, we demonstrate that NODIII mouse isolated uterus displays a weak spontaneous motility in terms of both force of contraction and peak frequency. To further investigate the nature of changes driven by the high glucose blood levels on the control of uterine tone, we used several well-known uterotonic endogenous agents, i.e., Ach, Oxy, or PGF2α. As expected, an NODIII isolated uterus displays a generalized impairment to all three agents tested. This finding is consistent with the clinical evidence reporting that diabetic women require higher doses of Oxy to induce or augment labor [46,47]. The role of the second messengers cAMP and cGMP in the regulation of myometrial function has been widely demonstrated [48,49]. It is well recognized that both cAMP and cGMP are involved in the maintenance of uterine homeostasis [49,50]. The uncontrolled hyperglycemia leads to a two-fold elevation of both cAMP and cGMP in uterus harvested from NODIII mice. This alteration of cAMP and cGMP levels in uterine tissue contributes to the unbalance of the uterine functionality.

We have previously reported that H_2_S pathway is involved in uterus homeostasis in physiological condition where a significant role is played by CSE-derived H_2_S [31]. It is of particular interest the finding that in NODIII mice the amount of H_2_S is 2-fold higher as compared to control mice. The intracellular cyclic nucleotides levels are controlled by the action of specific phosphodiesterases (PDEs) that rapidly hydrolyze the nucleotides. H_2_S acts as an endogenous nonspecific inhibitor of PDE activity, thereby enhancing cGMP levels [51,52]. Indeed, H_2_S increases cGMP levels in mouse aorta and can inhibit both cGMP and cAMP breakdown into a cell-free system [51]. Thus, within the diabetic uterine tissue, the increased endogenous H_2_S levels contribute to the shift toward a relaxing tone by elevating cGMP and cAMP. Among the three enzymes deputed to the biosynthesis of H_2_S within the uterus, the expression of CSE and CBS is reduced, while the 3-MST expression is significantly increased, in NODIII mice. These data suggest that within the diabetic uterus a major role could be played by 3-MST derived H_2_S. To further address this issue, we performed a pharmacological modulation study in vitro by using the isolated uterus and the enzymes’ endogenous substrate, i.e., L-Cys. It is important to note that the basal production of H_2_S in NODIII uterus homogenates is already 2-fold higher than the matched value of normoglycemic mice and that the incubation with L-Cys leads to a significant increase of about 45% as compared to the unstimulated uterus samples. Interestingly, in normoglycemic mice, the increase in L-Cys-induced H_2_S is higher than NOD mice reaching a 2-fold increase as compared to control values. As expected, the treatment with CSE or/and CBS inhibitors causes a significant reduction in H_2_S production in CTR mice. When the NODIII uterine tissues are incubated with L-Cys in presence of either CSE or CBS inhibitors or their combination, H_2_S levels are not significantly modified. These data may indicate that the source of H_2_S is mainly due to 3-MST, as also suggested by the western blot analysis. Thus, we hypothesize that 3-MST-derived H_2_S plays a major role in modulating uterus tone in diabetic condition. The minor role for H_2_S derived CBS/CSE obtained by the enzymatic activity evaluation in NODIII mice is confirmed by the functional studywhere L-Cys-induced relaxation is markedly impaired in NODIII mice. Thus, the data so far indicate that in NODIII mice uterus, there is a disruption of the endogenous H_2_S pathway leading to an increase in 3-MST-derived H_2_S that in turn elicits an increase in cAMP and cGMP. However, if these endogenous changes lead to alterations in the uterus response to exogenous H_2_S, it is an important issue to address. The exposure of an isolated uterus to NaHS shows a similar profile between NODIII and CTR mice indicating that the dysregulation of the H_2_S pathway within the uterus leads also to a tolerance to exogenous H_2_S response as opposed to what happens within the vasculature. Indeed, isolated aorta rings harvested by NODIII mice display a reduced response to L-Cys coupled to an increased relaxation to NaHS in presence of an over-expression of CBS and CSE [12].

In conclusion, in NODIII mice, the increase in H_2_S levels within the uterus is mainly 3-MST-derived, and it contributes to the shift toward a relaxing tone leading to a reduction in the spontaneous endogenous contractions. This disrupted environment translates into a generalized hypo-contractility to stimuli, such as PGF2α and Oxy, that are also clinically used to induce labor. A better understanding of the role played by the H_2_S pathway may lead to define novel therapeutic targets.
